# An Autosomal Translocation 73,XY,t(12;20)(q11;q11) in an Infertile Male Llama (*Lama glama*) With Teratozoospermia

**DOI:** 10.3389/fgene.2019.00344

**Published:** 2019-04-16

**Authors:** Malorie P. Baily, Felipe Avila, Pranab J. Das, Michelle A. Kutzler, Terje Raudsepp

**Affiliations:** ^1^School of Veterinary Medicine, University of California, Davis, Davis, CA, United States; ^2^ICAR-National Research Centre on Pig, Assam, India; ^3^Department of Animal and Rangeland Sciences, College of Agricultural Science, Oregon State University, Corvallis, OR, United States; ^4^Department of Veterinary Integrative Biosciences, College of Veterinary Medicine and Biomedical Sciences, Texas A&M University, College Station, TX, United States

**Keywords:** camelids, cytogenetics, translocation, FISH, fertility, teratozoospermia

## Abstract

Structural chromosome abnormalities, such as translocations and inversions occasionally occur in all livestock species and are typically associated with reproductive and developmental disorders. Curiously, only a few structural chromosome aberrations have been reported in camelids, and most involved sex chromosomes. This can be attributed to a high diploid number (2n = 74) and complex chromosome morphology, which makes unambiguous identification of camelid chromosomes difficult. Additionally, molecular tools for camelid cytogenetics are sparse and have become available only recently. Here we present a case report about an infertile male llama with teratozoospermia and abnormal chromosome number 2n = 73,XY. This llama carries an autosomal translocation of chromosomes 12 and 20, which is the likely cause of defective spermatogenesis and infertility in this individual. Our analysis underlines the power of molecular cytogenetics methods over conventional banding-based chromosome analysis for explicit identification of normal and aberrant chromosomes in camelid karyotypes. This is the first case of a translocation and the first autosomal aberration reported in any camelid species. It is proof of principle that, like in other mammalian species, structural chromosome abnormalities contribute to reproductive disorders in camelids.

## Introduction

Numerical and structural chromosome abnormalities are well-documented causes of congenital abnormalities and reproductive disorders in all livestock species (reviewed by Villagomez et al., 2009; Raudsepp and Chowdhary, 2016; Szczerbal and Switonski, 2016). Aberrations such as aneuploidies, deletions and duplications result in genetic overdose or haploinsufficiency, and may severely affect viability, development and/or reproduction. Translocations and inversions, on the other hand, are often balanced and do not cause loss or gain of the genetic material. Consequently, phenotypic effects of balanced rearrangements may not be so obvious regarding the viability and appearance of the carrier. However, balanced structural rearrangements affect meiosis and gametogenesis, resulting in reduced fertility or infertility (Villagomez et al., 2009; Ghosh et al., 2016; Raudsepp and Chowdhary, 2016). Regardless, chromosome abnormalities are of concern in all livestock species and cytogenetic analysis is a routine approach for evaluating breeding animals and for testing animals with reproductive or developmental problems (Villagomez et al., 2009; Ducos et al., 2008; Lear and Bailey, 2008; Raudsepp and Chowdhary, 2016; Szczerbal and Switonski, 2016).

Compared to other domesticated species, clinical cytogenetics in alpacas, llamas and other camelids has progressed slowly. The first description of camelid karyotypes 50 years ago showed that all species have the same diploid number (2n = 74) with essentially similar chromosome morphology (Taylor et al., 1968). Since then, only a handful of reports have been published on chromosome aberrations in llamas and alpacas (reviewed by Raudsepp, 2014). These include only sex chromosome aneuploidies: two cases of X-monosomy (Hinrichs et al., 1997; Tibary, 2008), one case of X-trisomy (Tibary, 2008), two cases of XX female-to-male sex reversal (Wilker et al., 1994; Drew et al., 1999), and a dozen cases of XX/XY blood chimerism (Fowler, 1990; Hinrichs et al., 1997, 1999). So far, only two structural abnormalities have been described in camelids. One is the *Minute Chromosome Syndrome*, which has been found in infertile female alpacas and llamas (Drew et al., 1999; Tibary, 2008; Avila et al., 2014b; Fellows et al., 2014) and involves the smallest autosome, chromosome 36 (Avila et al., 2015). The second is an autosomal translocation in an infertile male llama that has been briefly and incompletely described in a study, whose main goal was the development of molecular cytogenetics tools for camelids (Avila et al., 2014b).

Reasons for the few clinical cytogenetics studies in camelids include a high chromosome number, a difficulty to identify chromosomes by conventional cytogenetic methods (Di Berardino et al., 2006; Avila et al., 2014b; Raudsepp, 2014), and the slow development of molecular cytogenetics tools for chromosome identification by fluorescence *in situ* hybridization (FISH) using DNA markers. While FISH has been a regular part of cytogenetics since the 1990s in most livestock/domestic species (Rubes et al., 2009), molecular cytogenetics tools for camelids became available only recently (Avila et al., 2014a,b, 2015).

Here, we revisit the prior partially studied case of the infertile male llama with an autosomal translocation (Avila et al., 2014b) and characterize it in detail clinically and cytogenetically using advanced semen imaging and conventional and molecular cytogenetic methods. This is the first autosomal translocation found in any camelid species.

## Materials and Methods

### Ethics Statement

Procurement of blood and tissue samples followed the United States Government Principles for the Utilization and Care of Vertebrate Animals Used in Testing, Research and Training. The protocols were approved by Institutional Animal Care and Use Committee as AUP #2009-115, AUP #2018-0342CA and CRRC#09-47 at Texas A&M University and ACUP #3817 at Oregon State University.

### Case Description

A physically normal male llama born in 2000 was referred for andrological and cytogenetic evaluation due to infertility. At the time of referral, the animal was 3 years old and had never sired a cria, despite multiple breeding attempts to different fertile females.

### Clinical Examination

Scrotum and testes were palpated for consistency to ensure that no gross pathology was present in the external genitalia. Testicular size was measured mechanically with sliding calipers. Testes and accessory glands were imaged with a real-time ultrasound scanner using a 5 MHz probe (Sonovet SV600, Universal Medical Systems Inc.) and testicular blood flow was measured in the marginal (MA) and supratesticular arteries (TA) by Doppler ultrasonography with an L12-5 probe (Kutzler et al., 2011).

### Semen Analysis

Semen was collected for six days using a ruminant artificial vagina inserted into a custom-designed llama phantom. Slides were prepared from each ejaculate using eosin-nigrosin staining method (Murcia-Robayo et al., 2018). Sperm morphology was first examined under a light microscope at 1000 x magnification, followed by transmission electron microscopy (TEM) with FEI TITAN 80–200 TEM/STEM with Chem-iSTEM Technology following standard procedures (Alvarenga et al., 2000; Pesch et al., 2006).

### Cell Cultures, Chromosome Preparation and Karyotyping

Samples for karyotyping included peripheral blood in Na-heparin (Becton Dickinson) as well as an ear clip in sterile Hank’s balanced salt solution containing 1X Antibiotic-Antimycotic solution (Gibco). The ear clip was used to establish primary fibroblast cultures. Metaphase chromosome preparations were obtained from short-term blood lymphocyte cultures or skin fibroblast cultures, according to standard procedures (Raudsepp and Chowdhary, 2008; Avila et al., 2014b). Chromosomes were stained with Giemsa for initial counting. Refined chromosome analysis and karyotyping were carried out by GTG banding (Seabright, 1971). Images for 20 cells were captured for each technique using an Axioplan2 microscope (Carl Zeiss) and IKAROS (MetaSystems GmbH) software. Twenty cells were karyotyped and chromosomes were arranged into karyograms following the nomenclature proposed by Balmus et al. (2007) and adopted for the alpaca by Avila et al. (2014b).

### DNA Isolation and Analysis of Sex Chromosomes by PCR

Peripheral blood was collected in EDTA vacutainers (Becton Dickinson) and DNA was isolated with Gentra Puregene Blood Kit (Qiagen), following the manufacturer’s protocol. Genomic DNA was used as a template for PCR reactions with alpaca primers for the Y-linked *SRY* gene (F:5′-GTCAAGCGCCCCATGAATGC-3′; R: 5′- CGTAGTCTCTGTGCCTCCTC-3′; 170 bp) (Drew et al., 1999) and the X-linked androgen receptor (*AR*) gene (F: 5′- GCTTTCCAGAACCTGTTCCA -3′; R: 5′- GCCTCTGCTCTGGACTTGTG -3′; 204 bp).

### Fluorescence *in situ* Hybridization (FISH)

Painting probes generated from flow-sorted alpaca X and Y chromosomes (Avila et al., 2014b) were used to test the presence and integrity of sex chromosomes. The origin of the autosomal translocation was investigated through series of dual-color FISH experiments with chromosome-specific markers (BAC clones from CHORI-246 BAC library^1^ derived from the alpaca whole genome cytogenetic map (Avila et al., 2014a). BAC DNA isolation, labeling and FISH were performed following standard protocols (Raudsepp and Chowdhary, 2008; Avila et al., 2014b). Images for a minimum of 10 metaphase spreads were captured for each experiment and analyzed with a Zeiss Axioplan2 fluorescence microscope equipped with Isis Version 5.2 (MetaSystems GmbH) software.

## Results

### Clinical and Semen Analysis

On physical examination, no general or reproductive abnormalities were found in the male llama. The testicles were of normal palpable consistency and size (Table 1). Ultrasonographic imaging of the testes and accessory sex glands revealed no abnormalities, with both the prostate and bulbourethral gland showing a typical homogeneous echotexture. Testicular blood flow measurements were within normal range for camelids (Table 2; Kutzler et al., 2011).

**Table 1:** Testicular measurements.

Testis	Length, cm	Width, cm	Diameter, cm
Left	4.2	2.2	2.4
Right	4.5	2.0	2.5


**Table 2:** Testicular blood flow measurements by Doppler ultrasonography of the marginal (MA) and supratesticular arteries (TA).

Testis	MA PSV, cm/s	MA EDV, cm/s	MA RI	TA PSV, cm/s	TA EDV, cm/s	TA RI
Left	6.30	4.80	0.24	11.10	3.40	0.69
Right	9.30	7.80	0.16	15.80	5.40	0.66
Average	7.80	6.30	0.20	13.45	4.40	0.68


*PSV, peak systolic volume; EDV, end diastolic volume; RI, resistance index*.

**FIGURE 1:**
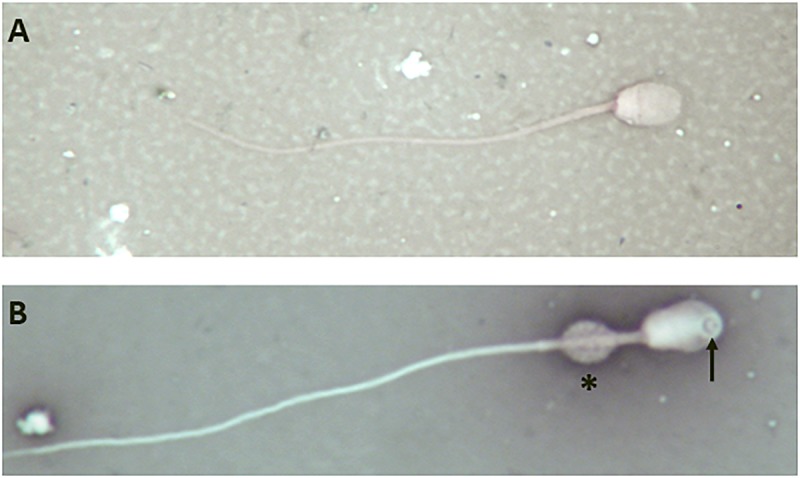
Light microscope images of eosin-nigrosine stained llama sperm. **(A)** Normal spermatozoon. **(B)** Abnormal spermatozoon showing a large nuclear vacuole (arrow) and an abnormally thickened midpiece (asterisk); magnification 1000×.

**FIGURE 2:**
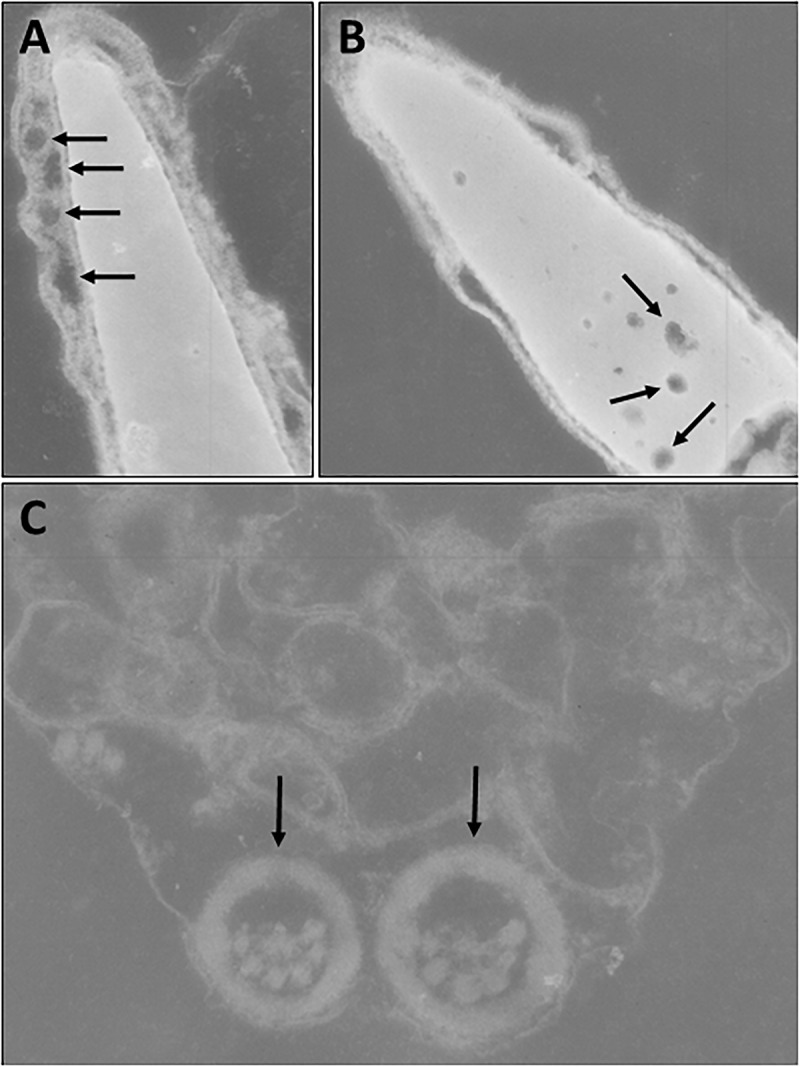
Transmission electron microscopy of sperm. **(A)** Abnormal spermatozoon with acrosomal vacuolation (arrows). **(B)** Abnormal spermatozoon with nuclear vacuolation (arrows). **(C)** Abnormal axoneme formation (arrows).

**FIGURE 3:**
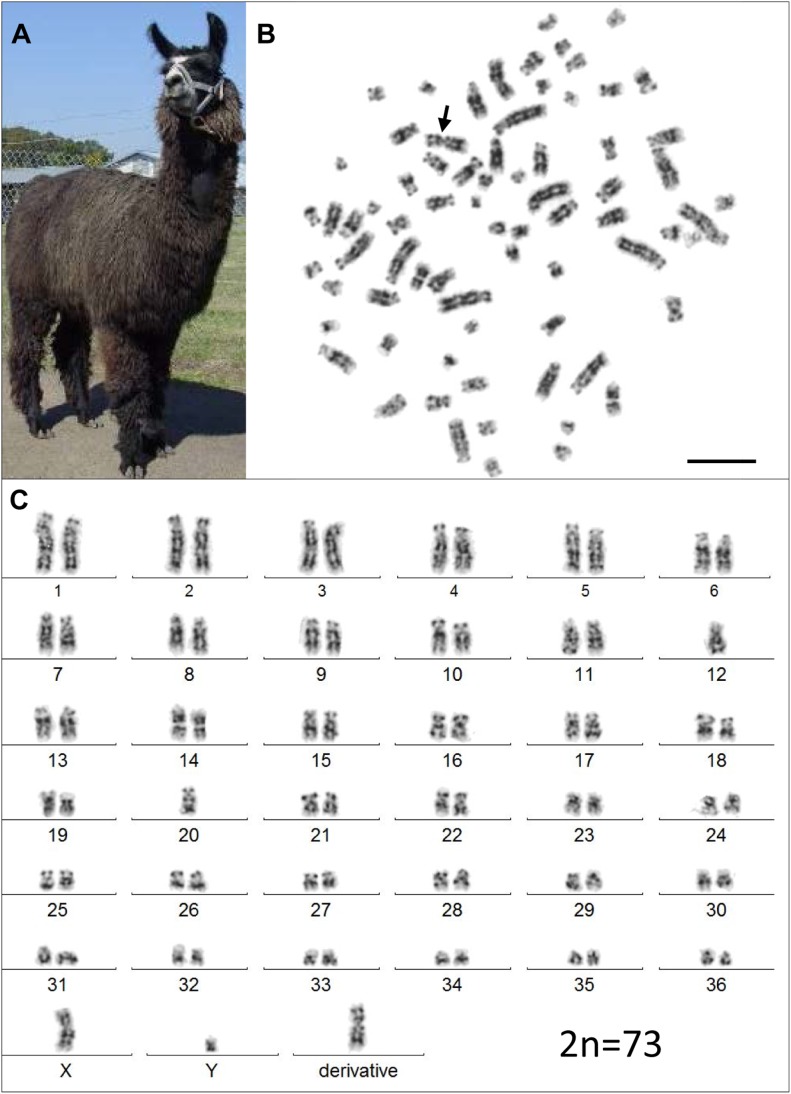
The infertile male llama and his karyotype. **(A)** The male llama showing overall normal appearance and viability. **(B)** A GTG-banded metaphase spread with the derivative chromosome (arrow). **(C)** Its corresponding karyotype (2n = 73). Bar 10 μm.

However, analysis of semen samples from six consecutive days, revealed that the percentage of morphologically normal sperm (Figure 1A) was low and ranged from 31.3 to 40.5% (Table 3). The most common abnormality observed was an abnormally thickened midpiece (Table 3 and Figure 1B), which occurred in 10.8–20.7% of the sperm evaluated. The occurrence of nuclear vacuolation was also high, ranging from 3.3 to 13.7% (Table 3 and Figure 1B). The semen abnormalities that were observed by light microscopic examination were confirmed and refined by TEM analysis. The latter showed the presence of both acrosomal (Figure 2A) and nuclear vacuolations (Figure 2B) as well as several sperm with abnormal axoneme formation (Figure 2C). Based on these findings, we concluded that the phenotypically normal-looking llama (Figure 3A) has severe teratozoospermia.

**Table 3:** Sperm morphology analysis results from daily semen collection.

Sperm Characteristic	Day 1	Day 2	Day 3	Day 4	Day 5	Day 6
Normal, %	40.5	31.3	35	33	36	34.9
Abnormally thickened midpiece, %	19.8	19.5	10.8	20.5	20.7	18.7
Detached head, %	6.6	15.2	16	10.2	13.7	9.7
Abnormally thick tail, %	0.82	0	0	0	0	0
Severely coiled tail, %	9.9	9.3	5.4	4.2	4.3	4
Abnormally long, skinny head, %	1.6	5.1	0	0	2.5	2.4
Short fat head, %	2.5	1.7	0	1.7	3.4	1.6
Bent midpiece, %	0	3.4	8.1	0	0.86	0
Severely bent midpiece, %	2.5	3.4	10.8	5.1	5.17	10.5
Microcephalia, %	9	2.5	5.4	4.2	6.8	7.3
Pyriform head, %	0.8	2.5	2.7	1.7	0.9	0.2
Broken midpiece, %	1.6	0.8	0	2.6	0	2.4
Nuclear vacuoles, %	3.3	3.3	5.4	13.7	5.19	4
Severely bent tail, %	0	0	0	0	0	1.6
Bent tail, %	0	0	0	0.8	0.86	0
Bent neck, %	0.8	1.7	0	0.8	1.7	0
Broken Neck, %	0	0	0	0	0	1.6
Total sperm counted	121	118	37	117	116	123


### Molecular Cytogenetic Analysis

Analysis of genomic DNA by PCR showed that the llama was positive for both the *SRY* and the *AR* genes, the expected profile of normal males.

**FIGURE 4:**
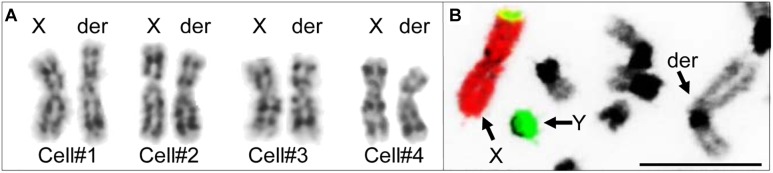
Investigating the origin of the derivative chromosome. **(A)** Comparison of GTG-banded chrX and the derivative chromosome in four different cells. **(B)** FISH with alpaca chrX (red) and chrY (green) painting probes showing non-sex chromosomal origin of the derivative chromosome (modified from Avila et al., 2014b). Bar 10 μm.

**FIGURE 5:**
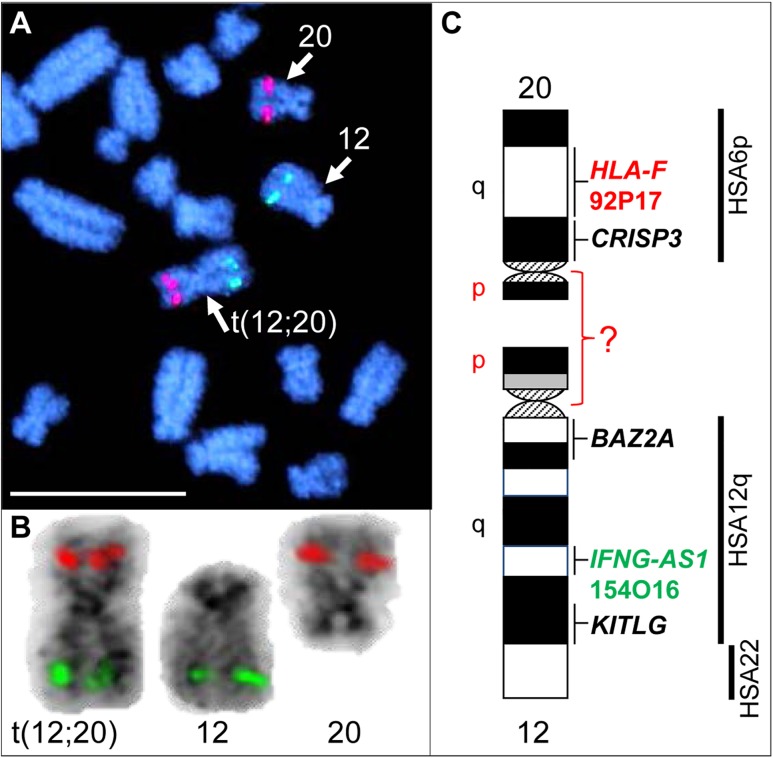
FISH with chr12 (green; BAC 154O16; *IFNG-AS1*) and chr20 (red; BAC 92P17; *HLA-F*) specific probes identifies the origin of the derivative chromosome. **(A)** Partial metaphase showing the location of normal chr12, normal chr20 and their translocation product. **(B)** Derivative chromosome, normal chr12 and normal chr20 (inverted) with corresponding FISH signals. **(C)** Schematic G-banded ideograms of chr12 and chr20 with information about mapped genes and human (HSA) homology (far right). Bar 10 μm.

Initial karyotyping of Giemsa-stained chromosomes indicated an abnormal diploid number of 2n = 73 in all metaphase spreads studied. This was confirmed via refined analysis by GTG banding, which also revealed the presence of a large submetacentric derivative chromosome (Figure 3B,C) that resembled the X chromosome in size, morphology, and banding pattern (Figure 4A). However, FISH analysis with alpaca X and Y chromosome painting probes showed that all cells contained only one X and one Y chromosome (Figure 4B), thus ruling out a sex-linked origin for the derivative chromosome. This suggested that the derivative chromosome was a result of a translocation of two medium-sized autosomes, which also explained the abnormal chromosome number of 73. Attempts to identify the autosomes involved in translocation by GTG banding were inconclusive due to morphological and banding similarities amongst different chromosome pairs in camelids (Figure 3C; Balmus et al., 2007; Avila et al., 2014b). Therefore, we conducted multiple dual-color FISH experiments with pairs of alpaca BAC clones containing markers specific to likely candidate chromosomes for the aberration. This analysis revealed that the derivative chromosome was the result of a translocation between chr12 and chr20 (Figure 5A,B). The markers that identified the derivative chromosome were BACs 4J13 and 154O16 for chr12 and BAC 92P17 (Figure 5C) for chr20 (Avila et al., 2014a). The karyotype of the infertile male llama was denoted as 73,XY,t(12;20).

## Discussion

The case of an autosomal translocation in an infertile male llama described herein is the first report of a translocation in any camelid species.

While FISH results demonstrated that the derivative chromosome harbors the long arms (q-arms) of chr12 and chr20 (Figure 5), we could not trace the location of the short arms (p-arms) of these chromosomes because no DNA markers have been, as yet, mapped to these regions (Avila et al., 2014a). On the other hand, if the fusion occurred between chr12p and chr20p (Figure 5C), additional rearrangements had to have taken place to define the centromere of the derivative chromosome. Thus, we consider the fusion of short arms unlikely. It is more plausible that the derivative chromosome resulted from a centric fusion of chr12q and chr20q, with subsequent loss of 12p and 20p. This is in keeping with observations that the short arms of most camelid chromosomes are heterochromatic and vary in size between individuals, as well as between homologs as shown by C-banding (Bianchi et al., 1986; Di Berardino et al., 2006; Balmus et al., 2007; Avila et al., 2014b). The fact that gene sequences have been assigned to the long arms of all 36 alpaca autosomes, but only to short arms of 6 of these (Avila et al., 2014a), further suggests their heterochromatic nature. It must be noted that because C-banding does not allow chromosome identification in camelids, it was not possible to evaluate heterochromatin in chr12p and 20p by this method. Attempts to combine C-banding with FISH for chromosome identification were also unsuccessful. However, heterochromatin at camelid centromeres and chromosome arms is AT-rich and stains bright with DAPI (black with inverted DAPI), which is the case with chr12p and chr20p (Figure 5B). Therefore, we theorize that the translocation did not cause loss of functionally important genetic material in somatic cells and can be considered as a balanced translocation. This is consistent with the overall normal appearance and viability of the carrier llama (Figure 3A).

Balanced translocations typically disturb meiotic pairing and segregation, resulting in the production of both genetically balanced and unbalanced gametes (Ghosh et al., 2016; Raudsepp and Chowdhary, 2016). The latter, if involved in fertilization, will cause embryonic death and, thus, subfertility of the translocation carrier. Such cases have been abundantly described in all livestock species (Ghosh et al., 2016; Raudsepp and Chowdhary, 2016; Szczerbal and Switonski, 2016). The llama in the present study has a more pronounced abnormal reproductive phenotype (teratozoospermia and sterility) than cases previously described in other livestock species, suggesting that the translocation may have disrupted function of genes important for normal spermatogenesis and/or fertilization. For example, camelid chromosome 20 is homologous to human (HSA) chromosome 6p and harbors the major histocompatibility complex (MHC) and a cluster of genes encoding for cysteine rich secretory proteins – the *CRISP* genes (Avila et al., 2014a). Of these, *CRISP3* was cytogenetically mapped very close to the presumed translocation break/fusion point (Figure 5C). CRISP proteins are expressed in the male reproductive tract and have known roles in sperm function, sperm-egg interactions, and overall fertility in many mammalian species, including camelids (Waheed et al., 2015; Gottschalk et al., 2016; Fedorka et al., 2017). The counterpart of the translocation, chr12, is homologous to HSA12q and part of HSA22 (Balmus et al., 2007; Avila et al., 2014a; Figure 5C), but no male fertility genes have been mapped to this camelid chromosome. Therefore, even though the involvement of *CRISP3* in the translocation is an appealing target for speculation, the molecular consequences of this rearrangement remain unknown and will be particularly interesting for follow-up.

In summary, we described the first chromosomal translocation in camelids, its likely causative relationship with teratozoospermia and infertility, and demonstrated the power of molecular cytogenetic approaches for the detection of structural aberrations in species with complex karyotypes. Characterization of molecular and functional consequences of such rearrangements, however, requires further research and improved knowledge about camelid genomes.

## Author Contributions

MK and TR initiated and designed the study. MB, FA, MK, PD, and TR conducted experimental work and data analysis. MB, FA, and TR wrote the manuscript with input from all authors.

## Conflict of Interest Statement

The authors declare that the research was conducted in the absence of any commercial or financial relationships that could be construed as a potential conflict of interest.

## Footnotes

1 https://bacpacresources.org/
